# Autoimmune Encephalitis: A Physician’s Guide to the Clinical Spectrum Diagnosis and Management

**DOI:** 10.3390/brainsci12091130

**Published:** 2022-08-25

**Authors:** Arpan Patel, Yue Meng, Amanda Najjar, Fred Lado, Souhel Najjar

**Affiliations:** 1 Department of Neurology, The University of Kansas School of Medicine, The University Kansas Medical Center, 3599 Rainbow Boulevard, Kansas City, KS 66103, USA; 2 Stanford University School of Medicine, Stanford Health Care, Palo Alto, CA 94305, USA; 3 Ferkauf Graduate School of Psychology, Yeshiva University, Bronx, NY 11549, USA; 4 Department of Neurology, Zucker School of Medicine at Hofstra/Northwell, New York, NY 11549, USA

**Keywords:** autoimmune encephalitis, encephalitis symptoms, anti-NMDA encephalitis, encephalitis, paraneoplastic, diagnosis autoimmune encephalitis, treatment autoimmune encephalitis, pathogenesis autoimmune encephalitis, review autoimmune encephalitis

## Abstract

The rapidly expanding spectrum of autoimmune encephalitis in the last fifteen years is largely due to ongoing discovery of many neuronal autoantibodies. The diagnosis of autoimmune encephalitis can be challenging due to the wide spectrum of clinical presentations, prevalence of psychiatric features that mimic primary psychiatric illnesses, frequent absence of diagnostic abnormalities on conventional brain MR-imaging, non-specific findings on EEG testing, and the lack of identified IgG class neuronal autoantibodies in blood or CSF in a subgroup of patients. Early recognition and treatment are paramount to improve outcomes and achieve complete recovery from these debilitating, occasionally life threatening, disorders. This review is aimed to provide primary care physicians and hospitalists who, together with neurologist and psychiatrists, are often the first port of call for individuals presenting with new-onset neuropsychiatric symptoms, with up-to-date data and evidence-based approach to the diagnosis and management of individuals with neuropsychiatric disorders of suspected autoimmune origin.

## 1. Introduction

Autoimmune encephalitis is among the most common form of encephalitis of non-infectious etiologies caused by autoantibodies targeting neural epitopes such as synaptic surface structures (e.g., receptors, ionic channels, or supporting proteins) or intracellular antigens such as onconeural antigens [1]. The rapidly expanding spectrum of autoimmune encephalitis in the last fifteen years is largely due to ongoing discovery of many neuronal autoantibodies. According to a recent epidemiologic study, the current prevalence and incidence of autoimmune encephalitis are estimated to be comparable to those of all infectious encephalitis, with rapidly increasing rates of detection [2]. Autoimmune encephalitis might etiologically account for a subgroup of illnesses previously classified as cryptogenic encephalitis, intractable epilepsy, atypical movement disorders, rapidly progressive dementia of unknown etiologies, or psychiatric and behavioral disturbances mistaken for primary psychiatric illnesses such as schizoaffective spectrum disorder and acute mania. Increased familiarity with the neurobiology and the diverse presenting neuropsychiatric symptoms of autoimmune encephalitis among physicians of all disciplines, and not only neurology, is pivotal for early detection. This is prognostically important, as early recognition and timely treatment can substantially improve the clinical outcome of these severely disabling, although often reversible, disorders, and can also lessen the financial burden associated with prolonged inpatient hospitalization due to delayed or inaccurate diagnosis [3]. This becomes more relevant in the view of the emerging data, suggesting that the demand for neurologists continues to outgrow the supply worldwide [4].

This review aims to provide primary care physicians and hospitalists who, together with neurologists and psychiatrists, are often the first port of call for individuals presenting with new-onset neuropsychiatric symptoms, with up-to-date data and evidence-based approach to the diagnosis and management of individuals with neuropsychiatric disorders of suspected autoimmune origin.

## 2. What Is Autoimmune Encephalitis?

Encephalitis is a complex heterogeneous inflammatory syndrome of diverse etiologies (infectious, inflammatory, or immunological) affecting the brain parenchyma [5,6]. In immune-mediated encephalitis, the body’s immune system identifies specific nervous system epitopes as immunological targets and mounts an immune response against them. Immune-mediated encephalitis can be etiologically linked to systemic inflammatory autoimmune disorders diseases such as systemic lupus erythematous, post-infectious processes such as post-viral acute demyelinating encephalomyelitis (ADEM), and post herpes simplex virus anti N-methyl-D-aspartate receptor (NMDAR) encephalitis [7] or paraneoplastic autoimmunity. However, it can also be idiopathic where no clear primary immunological trigger is identified. The target antigens in autoimmune encephalitis are either neuronal cell surface proteins and synaptic receptors such as NMDA and γ-aminobutyric acid (GABA) receptors or intracellular epitopes such as onconeural antigens (e.g., Hu and Ma2) and glutamic acid decarboxylase (GAD) [8].

**Figure 1:**
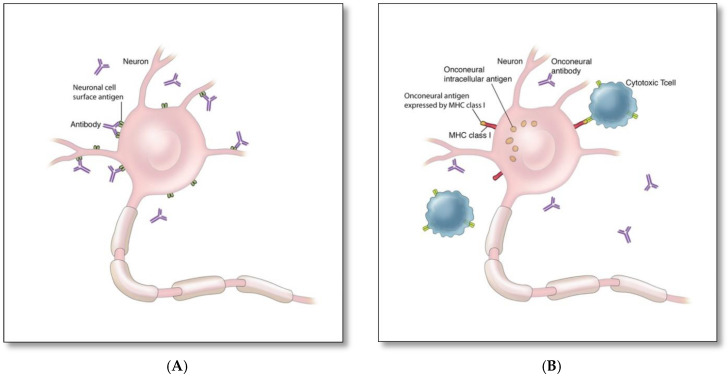
Mechanisms of autoimmune encephalitis: (**A**) Autoimmune encephalitis associated with antibodies against synaptic surface proteins (e.g., NMDAR, LGI1, AMPAR) [8], (**B**) Autoimmune encephalitis associated with antibodies against intracellular cytoplasmic (e.g., Yo, CV2/CRMP5) or nuclear (e.g., Hu, Ri) onconeural antigens [9,10,11].

**Figure 2:**
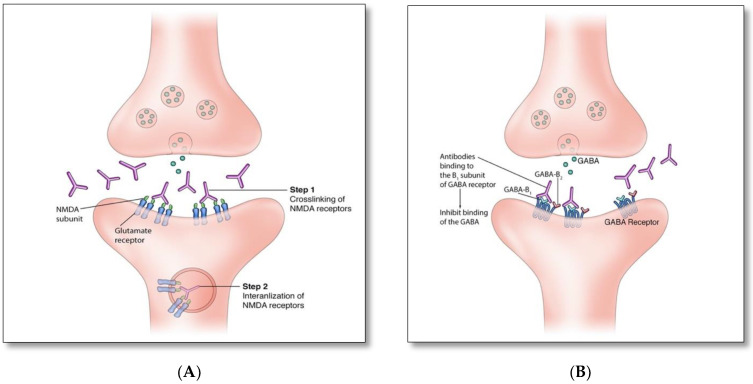

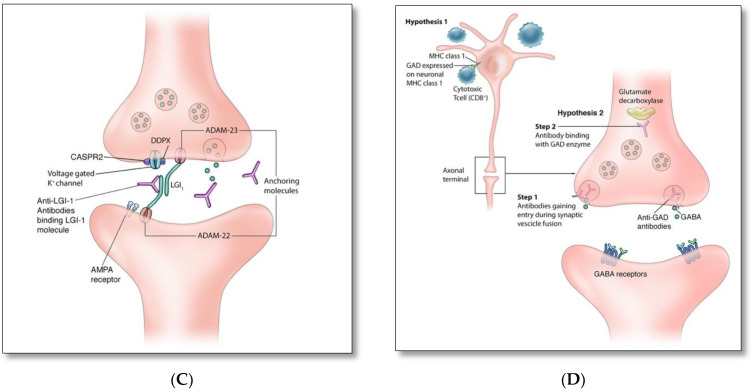
(**A**). Anti-NMDAR antibodies bind to an extracellular GluN1 subunit of NMDAR and reduce NMDAR density by cross-linking and internalizing these receptors. (**B**). Similar to anti-GABA_B,_ anti-Glycine, -dopamine-2R, -AMPA, and -GABA_A_ receptor antibodies act through blocking receptors and directly preventing neurotransmitter binding to the receptor. (**C**). Disruption of channel opening and blocking function of supporting proteins: Anti-VGKC, -CASPR2, and -DDPX antibodies interfere with the opening of potassium channels and the release of Glutamate; anti-LGI1 antibodies block the binding of LGI1 with ADAM22 and ADAM23, which indirectly interferes with AMPAR function. (**D**). Intracellular synaptic protein: two proposed mechanisms of anti-GAD associated autoimmunity [8,14,15,16].

There are two proposed immune mechanisms in autoimmune encephalitis [9,10,11,12,13]. The first involves autoantibodies against synaptic surface structures (e.g., receptors, ionic channels, or supporting proteins) (Figure 1A) [8]. These antibodies cause neuronal dysfunction by altering synaptic transmission through cross-linking and internalization of the receptors such as anti-NMDAR antibodies (Figure 2A), preventing neurotransmitter binding such as anti-GABA_B_ receptor antibodies (Figure 2B), or disrupting ion channel function such as anti-VGKC and anti-LGI1 antibodies (Figure 2C) [8]. Thus, the clinical outcome is generally favorable if diagnosed and treated early, as these antibodies do not directly damage neuronal structures or produce significant neuronal apoptosis cell bodies in early phase [12,13].

The second mechanism involves cytotoxic T cell-mediated neuronal destruction associated with antibodies against cytoplasmic (e.g., Yo, CV2/CRMP5) or nuclear onconeural antigens (e.g., Hu, Ri) (Figure 1B) [9,10,11]. These antibodies are not directly harmful, but rather serve as biomarkers for the concurrent pathogenic cytotoxic T cell-mediated autoimmunity [9,10,11], which is typically associated with limited response to treatment and worse neurological outcome due to rapid neurodegeneration, despite treatment [8].

It is unclear whether humoral (Figure 1A) or cytotoxic (Figure 1B) mechanisms account for the pathogenesis of GAD autoantibodies associated autoimmunity. GAD is an intracellular synaptic enzyme that mediates glutamate conversion into GABA (Figure 2D) [14,15]. Intrathecal synthesis of GAD autoantibodies is well demonstrated using cerebrospinal fluid (CSF) studies; however, it remains unknown whether these antibodies can cross axonal terminal membranes and bind intracellularly to GAD [14]. It is postulated that the pathogenicity associated with these autoantibodies is indirectly related to the concurrent cytotoxic T cell-mediated autoimmunity [15]. Alternatively, it is possible that these antibodies may gain entry into the axonal terminal during synaptic vesicle fusion associated with presynaptic neurotransmitter release and binds to GAD enzyme, similar to that associated with amphiphysin autoantibodies [16].

## 3. History of Autoimmune Encephalitis

Our knowledge of autoimmune encephalitis began as early as 1938, when Brouwer and Biemond reported a patient with cerebellar degeneration and ovarian cancer [17]. They postulated that cerebellar degeneration can be associated with cancer located elsewhere in the body and small in size [17]. Russell was the first to propose a mechanism of neurological injury in paraneoplastic syndromes. He described patients with encephalomyelitis and sensory neuropathy and attributed the neurological symptoms to the formation of autoantibodies triggered by exposure of the immune system to certain cancers [18]. In 1968, Corsellis and colleagues described patients with lung cancer who also developed memory loss, neuropsychiatric disturbances, and seizures—without evidence of brain metastases [19]. At autopsy, the investigators found significant inflammatory involvement of limbic areas and termed this new entity ‘limbic encephalitis’. Corsellis and colleagues understood limbic encephalitis to be a rare disorder, associated with end-stage complications of cancer [19]. In 1981, Newsom-Davis established an autoimmune mechanism for paraneoplastic neurological disorder by successfully treating three cases of Lambert–Eaton myasthenic syndrome with plasma exchange and immunosuppression with steroids [20].

Many classic paraneoplastic neurological syndromes associated with onconeural autoimmune antibodies were discovered from 1980 to 2000, including anti-Hu, -Yo, -Ma, -Amphiphysin, and -Tr [21]. In 1990, Posner compared serum and CSF profiles of 18 patients before and after plasmapheresis, concluding that autoantibodies in paraneoplastic syndrome may be synthesized in the CSF [22]. In 2001, Ligouri et al. and Buckley et al. independently described case reports of two individuals with plasmapheresis responsive limbic encephalitis associated with voltage-gated potassium channels (VGKC) antibodies [23,24].

**Figure 3:**
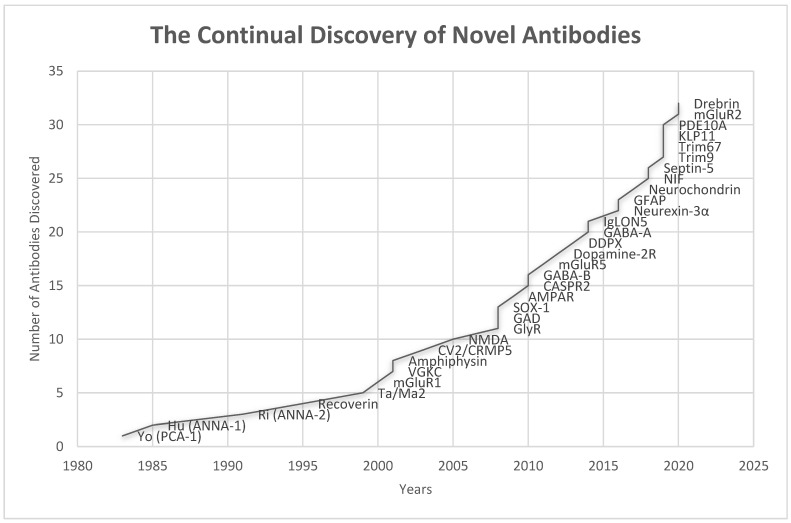
Timeline of the discoveries of various antibodies associated with autoimmune encephalitis (Anti-GAD was discovered in 1988 in patient with stiff person syndrome, but its association with autoimmune encephalitis was not known until 2008) [23,25,32,33,34,35,36,37,38,39,40,41,42,43,44,45,46,47,48,49,50].

Dalmau and colleagues described the first case of encephalitis associated with NMDAR autoantibodies in 2005, and later published a case series of 12 cases of encephalitis associated with these autoantibodies presenting with memory impairment, seizures, and neuropsychiatric symptoms [25,26,27]. Initially, this neurological entity was referred to as paraneoplastic encephalitis that responded to immunotherapy and oophorectomy [27]. This clinical syndrome is subsequently referred to as an “autoimmune encephalitis” to encompass the full spectrum of encephalitides irrespective of their association with the cancer [25,26,27], given that the majority of central nervous system (CNS) autoimmunity attributed to autoantibodies targeting neuronal surface antigens and synaptic proteins are not associated with tumors [12]. Since 2007, many new antibodies targeting neuronal surface antigens and synaptic proteins causing new forms of autoimmune encephalitis have been discovered, including those caused by autoantibodies targeting GABA_B_-R, GABA_A_-R, α-amino-3-hydroxy-5-methyl-4-isoxazolepropionic acid receptor (AMPAR), and contactin-associated protein-like 2 (CASPR2), and the list continues to grow (Figure 3). In 2011 Najjar and colleagues, along with other investigators introduced the term “seronegative autoimmune encephalitis” to highlight a group of immune–responsive encephalitic disorders without identifiable IgG-class neuronal autoantibodies in the serum [28,29]. In 2015, Armangue et al. described autoimmune encephalitis triggered by herpes simplex (HSV) encephalitis in 12–27% patients with HSV [30]. In 2016, it was found that the class of “checkpoint inhibitor” cancer immunotherapy, which can activate the immune system by promoting T cell-mediated cancer cell destruction, might also cause autoimmune encephalitis and other paraneoplastic neurologic syndrome in a small fraction of patients [31].

## 4. Pathophysiology and Immune Triggers of Autoimmune Encephalitis

There is a consensus that circulating neuronal autoantibodies must gain access to the CNS through the tightly regulated blood-brain or blood-CSF barriers (BBB and BCSFB, respectively) to exert their harmful pathogenic effects. To date, however, the precise mechanisms permitting that access remain largely elusive. The recent discovery of the relationship between dual lymphatic channels and the brain’s ‘glymphatic’ system, where glia channel CSF flow through periarteriolar and parenchymal extracellular spaces which [51] allows small molecule passage (like CNS proteins), can act as intracranial antigen and challenge the widely held immune privilege status of the central nervous system [51]. Other possibilities include inflammation-induced hyperpermeability of BBB or BCSF that can expose and re-expose the brain’s self-antigens to the peripheral adaptive immunity, which in turn can trigger the formation of pathogenic neuronal autoantibodies, leading to a breakdown in immune tolerance [52]. Viral infections are thought to serve as immune triggers in some cases by promoting production of cross-reactive autoantibodies against neuronal self-antigens and facilitating their entry into the CNS through mechanisms involving proinflammatory cytokines [52], particularly interleukin-17 produced by a subset of T-helper lymphocytes referred to as T_h_17 cells, which have been implicated in numerous autoimmune brain disorders [53]. In animal models, T_h_17 are shown to damage multiple BBB components, including tight junctions [54]. Proinflammatory states can also induce microglial priming, leading to excessive microglial production of inflammatory mediators, such as matrix metalloproteinases and pro-inflammatory cytokines such as IL-6, IL-10, and CXCL-13, which may affect the integrity and increase BBB permeability [55,56].

BBB disruption associated with perivascular neuroinflammation involving both innate and adaptive immune cells has been documented in brain biopsies and post-mortem studies from individuals with etiologically diverse autoimmune encephalitis [57]. BBB breakdown can also increase crosstalk between the CNS innate immunity and peripheral adaptive immunity, which in turn can perpetuate harmful effects of neuroinflammation on the BBB [58]. Collectively, these findings support the potential contributory role of BBB disruption to the pathophysiology of autoimmune encephalitis [59]. However, it is not yet clear whether BBB disruption precedes or follows neuroinflammation.

## 5. Clinical Spectrum

The clinical presentations and features of autoimmune encephalitis are highly diverse and largely dependent on the particular neuronal antigens and specific brain regions targeted by the autoimmune process (Table 1). Clinical features include acute to subacute onset of rapidly progressive diverse neurological and neuropsychiatric symptoms that can include psychiatric and behavioral disturbances [60,61], cognitive dysfunction [62], involuntary movements [63], intractable seizures [64,65], sleep disturbances [66], autonomic instability, and decreased level of consciousness, among others (Table 1). The initial presentation, however, can be limited to a few or even isolated neurological or neuropsychiatric disturbances, which can also remain dominant throughout the clinical course of the illness. These presentations include intractable epilepsy [67], chronic encephalopathy masquerading as atypical neurodegenerative process [68], unexplained psychiatric symptoms [60,61], or an isolated neurological syndrome such as cerebellar syndrome or movement disorder.

Psychiatric presentations of autoimmune encephalitis are common [60,61]. Several retrospective studies have shown that more than 80% of patients with NMDA encephalitis initially presented with psychiatric symptoms requiring psychotropic medications and, in some instances, psychiatric hospitalization [85,86]. The range of psychiatric symptoms is broad and may include personality changes, bizarre behaviors, agitation, anxiety disorders, depressive or manic symptoms, auditory or visual hallucinations, delusions, or catatonia [85,86]. They can be mistaken for primary psychiatric illnesses such as new-onset primary psychosis, schizoaffective spectrum disorder, and acute mania [60,61]. Antipsychotics are often not effective and associated with higher incidence of significant side effects such as neuroleptic malignant syndrome [61,87]. A recently published position paper on autoimmune psychosis draws attention to the increasingly recognized cases of autoimmune encephalitis presenting with isolated psychosis (including delusion, hallucination, thought disorganization, agitation, and aggression) and provides an international consensus on an approach to the diagnosis and management of these patients [61].

Seizures, including clinical and electrographic seizures, are also common and can be the presenting symptom [67]. Seizures are either localization-related or secondarily generalized, including convulsive status epilepticus [67,88]. Seizures of immunological etiologies tend to be frequent, resistant to anti-epileptic medications, rapidly progressive, and often with comorbid progressive encephalopathy [88]. Certain seizure semiologies and EEG patterns are highly characteristic for specific autoimmune encephalitis: faciobrachial dystonic seizures—brief motor seizures lasting for 1–3 s typically affecting the limbs and face simultaneously—are highly characteristic of anti-LGI1 encephalitis [65,70].

Movement disorders are common in autoimmune encephalitis, particularly at a younger age. Their phenotypes are broad and include orofacial dyskinesia, paroxysmal dyskinesia, chorea (brief, irregular, non-purposeful movements), dystonia (involuntary contractions of groups of muscles causing repetitive movement and abnormal posture), myoclonic jerks, tremor, cerebellar ataxia, and parkinsonian symptoms [63]. The clinical phenotypes of movement disorders are partly dependent on the causative neuronal autoantibodies; orofacial dyskinesia are common in mid-late stages of anti-NMDAR encephalitis [69], Morvan syndrome (peripheral nerve hyperexcitability) manifesting as myokymia and neuromyotonia are characteristic of anti-CASPR2 encephalitis [89], hyperekplexia is commonly seen in anti-DPPX encephalitis [74], progressive encephalomyelitis with rigidity and myoclonus (PERM) is a typical presentation of anti-GlyR encephalitis [42], opsoclonus myoclonus is commonly seen in anti-GABA_B_ and anti-Ri encephalitis [34,73], and jaw dystonia, as well as laryngospasm, are also associated with anti-Ri encephalitis [34].

Some degree of cognitive impairment is the single most consistent finding in autoimmune encephalitis, such as those associated with autoantibodies targeting NMDAR, LGI-1, and GAD, among others [62,90,91,92]. However, mild forms of cognitive dysfunction can be overlooked in the presence of dramatic neuropsychiatric features such as psychosis, among others [93]. Cognitive impairment encompasses deficits in memory, language, executive functions, sustained attention, and apraxia [68]. Memory dysfunction typically manifests initially as a rapid decline in short-term memory and working memory. Long-term memory involvement is generally seen in either severe cases or those where treatment is delayed or inadequate. Language deficits and decreased verbal output are common, including comprehension difficulty and mutism reported in severe cases [93]. In the elderly, anti-NMDAR encephalitis can present with rapidly progressive dementia associated with profound memory decline [33]. Notably, early diagnosis is pivotal for cognitive recovery, as early treatment can reverse cognitive impairment completely or near completely even in severe cases, while delayed treatment may result in a persistent cognitive deficit [68].

Although sleep disorders are not listed as a diagnostic criterion in clinical diagnosis of autoimmune encephalitis [1], sleep disturbances are frequently observed, although at times overlooked, in individuals with autoimmune encephalitis [66]. A recent prospective study of 26 autoimmune encephalitis patients showed that 73% patients had new-onset sleep disruptions [66]. Common sleep disturbances include rapid eye movement (REM), sleep behavior disorder and dream enactment behaviors (e.g., anti-LGI1 and CASPR2 encephalitis) [94], hypersomnia and fragmented sleep (e.g., anti-Ma encephalitis) [95], insomnia and periodic limb movements (e.g., anti-NMDA encephalitis and anti-DPPX encephalitis) [74], and REM sleep behavior disorders with stridor and disordered breathing (e.g., anti-IgLON5 encephalitis) [75].

We have highlighted in Table 2 the “red flags” suggestive of a diagnosis of autoimmune encephalitis.

## 6. Diagnosis

**Figure 4:**
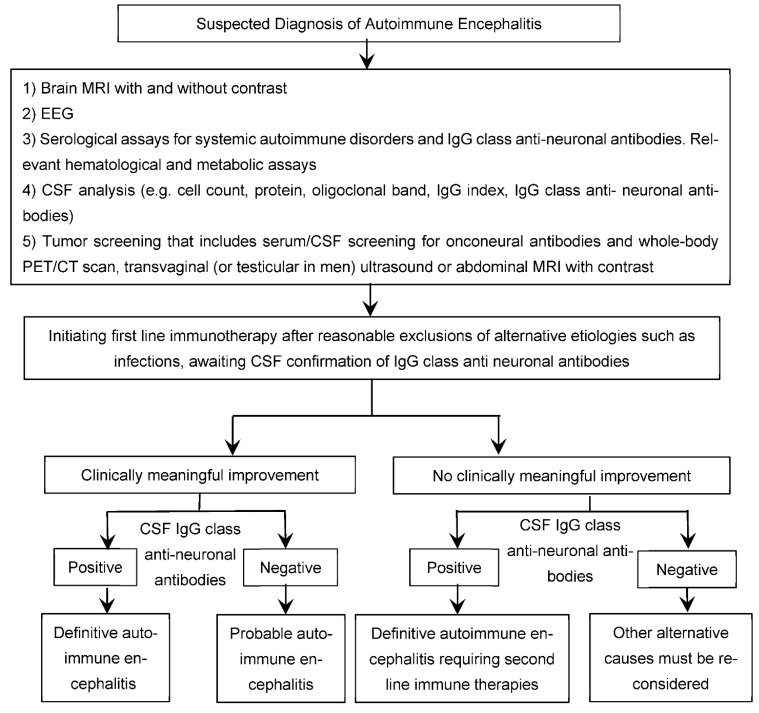
Suspected Diagnosis of Autoimmune Encephalitis.

The diagnosis of autoimmune encephalitis usually follows the 2016 autoimmune encephalitis criteria by Graus et al., as shown in Table 3 [1]. The diagnosis of autoimmune encephalitis can be challenging due to the wide spectrum of clinical presentations, prevalence of psychiatric features that mimic primary psychiatric illnesses, frequent absence of diagnostic abnormalities on conventional brain MR-imaging, non-specific findings on EEG testing, and the lack of identified IgG class neuronal autoantibodies in blood or CSF in a subgroup of patients [1,61]. Thus, the diagnosis requires a high index of clinical suspicion after reasonable exclusion of alternative causes and a lower threshold to test paired CSF/serum for confirmation of the presence of neuronal autoantibodies (Figure 4), particularly in those with red flags suggestive of underlying autoimmunity (Table 2). The challenge of establishing a diagnosis is greatest in those cases where neuronal autoantibodies are not identified in the CSF, despite a very high index of clinical suspicion and a reasonable exclusion of alternative etiologies including infectious etiologies (such as viral encephalitis, fungal infections, and tuberculosis) [96], rheumatologic causes (such as lupus and neuro-Behcet syndrome) [97], toxic-metabolic disturbances (such as substance abuse and Wernicke encephalopathy) [98], vascular disorders (such as reversible posterior leukoencephalopathy syndrome) [99], neoplastic diseases (such as leptomeningeal disease, diffuse glioma, and primary or secondary lymphoma), rapidly progressive neurodegenerative diseases (such as various causes of dementia and Creutzfeldt–Jakob disease) [100], primary psychiatric illnesses (such as schizophrenia and mood disorders) [60,61], Mitochondrial disorders (such as MELAS) [101], Metabolic diseases—Hashimoto encephalopathy, CNS vasculitis [1], and medication-related syndromes such as neuroleptic malignant syndrome and serotonin syndrome [98].

Diagnostic workup for individuals suspected to have autoimmune encephalitis:

(1) Screening of paired serum and CSF samples for the presence of IgG class anti-neuronal antibodies remains pivotal to the diagnosis of neuronal surface antibodies-associated autoimmune encephalitis [1,102]. However, many authors endorse cautious interpretation of results, revealing neuronal surface antibodies in only serum (particularly at low levels) and not CSF [1,102,103,104], with the exception of certain antibodies such as anti-LGI1 antibodies known to be more positive in serum than CSF [61]. CSF antibody assay appears to be more specific, consistent, and reliable than serum testing for autoimmune encephalitis arising from synaptic autoimmunity, such as anti-NMDA receptor encephalitis [1]. Detection of IgG class anti-neuronal antibodies in CSF is generally required for confirming the diagnosis of synaptic autoimmune encephalitis such as anti-NMDAR encephalitis [1,61]. Moreover, identification of specific neuronal surface antibodies can provide meaningful insight into the course, management, and prognosis of autoimmune encephalitis.

(2) Tumor screening: serum and CSF screening for antibodies and whole-body CT scan to search for occult malignancy [61]. Certain autoimmune encephalitis in which there is higher tumor association rate, should undergo further testing, such as whole body PET scan, transvaginal ultrasound, or abdominal MRI with contrast, or testicular ultrasound in men if initial tumor screening is negative.

(3) CSF biomarkers of inflammation or immune activation such as pleocytosis with more than five WBCs per mm^3^, oligoclonal bands, and elevated IgG index [1,21,105].

Of note, the prevalence of these abnormalities is higher among autoimmune reactions targeting NMDAR (90%), GABA_B_R (90%), and AMPAR (90%), as opposed to those mediated by LGI1 (40%) and CASPER2 (25%) [21,103,105,106,107].

(4) Serum biomarkers for systemic autoimmune disorders and Hashimoto’s encephalopathy in clinically relevant cases.

(5) Brain neuroimaging. Unilateral or bilateral hippocampal MRI FLAIR-T2 hyperintensities, with or without transient contrast enhancement, can be highly characteristic of paraneoplastic or non-paraneoplastic autoimmune limbic encephalitis [1,105,108]. By contrast, extra-limbic cortical and subcortical gray and white matter abnormalities are etiologically more heterogeneous than limbic involvement. However, conventional MRI is not a sensitive tool for diagnosing these patients [108,109], as brain structural abnormalities are often lacking on initial presentation and follow-up brain MRIs in about 89 and 79% of individuals with anti-NMDAR encephalitis, respectively, including those with profound neurological deficits [108].

(6) Brain 18Fluorodeoxyglucose (18F-FDG) positron emission tomography (PET) metabolic abnormalities are common but etiologically non-specific [1,103,110,111,112,113]. While cortical FDG hypometabolism is the most commonly observed pattern, FDG hypermetabolism might be more indicative of active and persistent neuroinflammatory process in the proper clinical context [60,103,114]. Clinical trials validating the usefulness of 18F-FDG PET imaging in autoimmune encephalitis are needed before incorporating it routinely into clinical practice [115].

(7) EEG abnormalities are common but not etiologically specific. These include slow-wave activity (focal or diffuse, rhythmic or polymorphic, symmetric or asymmetric, theta or delta), epileptiform discharges, and electrographic seizures [116,117,118]. Certain electrographic patterns are highly characteristic, such as extreme delta brushes (1–3 Hz slowing with superimposed 20–30 Hz activity) typically observed in about 30% of individuals with anti-NMDAR encephalitis [1,119].

(8) Clinically meaningful response to immunotherapy trials in patients with suspected autoantibody-negative psychosis of probable immune origin (Figure 4) might serve as circumstantial evidence of underlying immune dysregulation or autoimmunity [60]. However, it must be note that delayed clinical response to immunotherapies can limit its diagnostic utility [1]. Tissue-based assay can be used to confirm the diagnosis seronegative autoimmune encephalitis and facilitates initiation of immunotherapy.

(9) The diagnostic utility of brain biopsy is generally limited given the high sensitivity and specificity of recent immunological assays such as cell-based-assays [60,120]. However, given its low rate of complications [120], brain biopsy can be considered in selected cases of severe encephalitis of suspected autoimmune origin despite negative CSF assays for autoimmunity, including neuronal antibodies, in order to exclude alternative etiologies for inflammatory substrate, such as infections that can allow earlier initiation of aggressive immunosuppressive therapies [60].

## 7. Management

Although synaptic autoimmune encephalitis is often debilitating, and at times life-threatening, it is frequently reversible with a timely treatment with an effective immune therapy. The treatment recommendations for autoimmune encephalitis currently lack clinical trials or systemic reviews and are based solely on expert opinion and anecdotal evidence. Intravenous corticosteroids, intravenous immunoglobulin (IVIG), and plasma exchange are considered first line therapies (Table 4) [1,13]. It is generally recommended not to delay treatment with first-line immune therapies while awaiting paired CSF/serum confirmation of the presence of neuronal autoantibodies in individuals strongly suspected to have autoimmune encephalitis based on the typical clinical presentations and the paraclinical findings indicative of inflammatory processes (e.g., CSF pleocytosis and/or inflammatory changes affecting both mesial temporal lobe structures), after reasonable exclusion of alternative etiologies such as infections (Figure 4) [1,61,105]. This is also particularly relevant, with the rapidly emerging spectrum of encephalitic disorders strongly thought to be of immune origin by autoimmune encephalitis experts despite the lack of identified neuronal autoantibodies on paired serum and CSF assays (so-called neuronal autoantibody negative encephalitis) [1,21,61].

Second-line agents, including rituximab or cyclophosphamide, are considered for those who show inadequate response to first-line immune therapies or develop relapse despite appropriate maintenance therapy (Table 4) [13]. Rituximab combined with cyclophosphamide can provide greater therapeutic efficacy in highly selected severe forms of autoimmune encephalitis [121]. Tocilizumab and intravenous methotrexate can also be effective in severe forms of autoimmune encephalitis unresponsive to second-line immunotherapies (Table 4) [122,123,124]. Inebilizumab (humanized monoclonal antibody against the B-cell surface antigen CD19) is under clinical trial (ExTINGUISH Trial) for treatment of patients with NMDA encephalitis, and considered as one the emerging therapies for patients with autoimmune encephalitis [125]. Although no consensus guidelines exist, maintenance therapy for 1–2 years is generally recommended to prevent relapse (Table 4) [61,121]. Options for maintenance therapy include monthly IVIG infusions, high dose intravenous methylprednisolone pulse therapy, oral prednisone tapering, and steroid-sparing agents such as azathioprine and mycophenolate (Table 4) [121,126]. It must be emphasized that the recovery course is typically protracted, and that complete or near-complete recovery may take up to 2 years, despite timely treatment with proper immune therapies [127].

**Table 4:** Therapeutic options of autoimmune encephalitis [13,122,123,124,128].

Immunomodulator	Dosing Regimen
**First line immunomodulators**	
1. Pulsed methylprednisolone	1000 mg IV daily for 5 consecutive days
2. IVIG	2 g/kg bodyweight IV infusion typically divided over 5 days
3. Plasmapheresis	1 session every other day for an average of 5–7 sessions, based on response and tolerance
**Second line immunomodulators**	
1. Rituximab	Two 1000 mg doses separated by 2 weeks or weekly 375 mg/m^2^ infusions for 4 weeks
2. Cyclophosphamide	750–800 mg/m^2^ monthly for 3–6 months
**Third line immunomodulators**	
1. Tocilizumab	Initially 4 mg/kg, followed by an increase to 8 mg/kg monthly based on clinical response (maximum dose: 800 mg)
2. Intrathecal methotrexate	10–12 mg weekly for 3–4 weeks
**Maintenance therapy**	
1. IVIG	(Doses, frequency, and duration of treatment vary based on the symptom’s severity, relapse risk, and tolerance)
2. Pulsed methylprednisolone	
3. Oral prednisone	
4. Azathioprine	
5. Mycophenolate	

Patients usually need short-term treatment with anti-epileptic drugs for seizure control [64]. Management of co-morbid psychiatric manifestations such as depression, agitation, catatonia, bipolar disorders, and psychosis can be challenging [60,61]. For example, the use of antipsychotics to treat autoimmune encephalitis-associated psychosis is associated with a higher risk of autonomic instability and neuroleptic malignant syndrome [61,87]. Recommendations from a recent expert opinion-based international consensus on the management of the concurrent acute psychosis include using lower doses and slower titrations of atypical or second-generation antipsychotics and benzodiazepines in controlling agitation and catatonia [61].

## 8. Conclusions

The spectrum of autoimmune encephalitis continues to rapidly expand with the growing discovery of neuronal autoantibodies. Early recognition and treatment are paramount to improve outcomes and achieve complete recovery from these debilitating, occasionally life threatening, disorders. Early diagnosis, however, can be challenging due to the heterogeneity of the clinical presentations. It requires a high index of clinical suspicion and a low threshold for paired serum/CSF assays, particularly in the presence of clinical red flags suggestive of immune origin. This review aimed to provide primary care physicians and hospitalists who, together with psychiatrists, are often the first port of call for individuals presenting with new-onset neuropsychiatric symptoms, with an up-to-date data and evidence-based approach for the diagnosis and management of individuals with neuropsychiatric disorders of suspected autoimmune etiologies.

## Figures and Tables

**Table 1:** Clinical and immunologic features of common types of autoimmune encephalitis [8,61].

Targeted Antigens	Antigen Location	Patient Demographics; Median Age (Range); Male: Female Ratio	Frequency and Main Type of Associated Malignancy	Main Neurological and Psychiatric Presentations	Other Clinical Features
**NMDAR** [69]	Cell surface (extra-limbic cortices)	Young women and children; 21 yr (2 mo–85 yr); 1:4	Overall 40%; 58% in women 18–45 yr (teratoma)	Panencephalitis with psychiatric symptoms, behavioral changes; cognitive and short-term memory impairment, seizures, movement disorders (e.g., dyskinesias)	Catatonia, autonomic instability (50% has central hypoventilation)
**LGI1** [70]	Synaptic (limbic system)	Older men; 64 yr (31–84); 2:1	5–10% (thymoma)	Limbic encephalitis with short-term memory loss, seizures (particularly faciobrachial dystonic seizures)	Psychiatric symptoms such as depression, REM sleep behavior disorders, hyponatremia, rarely movement disorders (choreoathetosis, dyskinesia, dystonia)
**CASPR2** [71]	Synaptic	Older men; 66 yr (25–77); 9:1	Overall 20% (thymoma);	Morvan syndrome (peripheral nerve hyperexcitability and neuromyotonia), limbic encephalitis, sleep disorder, memory loss, dysautonomia, ataxia, neuropathic pain	Delusions and hallucinations, concurrent immune-mediated disorders (e.g., myasthenia gravis)
**AMPAR** [72]	Cell surface (limbic system)	Middle-aged women; 56 yr (23–81); 1:2.3	65% (thymoma, SCLC, or breast cancer)	Limbic encephalitis, encephalopathy with memory loss	Features of limbic encephalitis on MRI in about 67%; history of concurrent auto-immunity in about 50% [63]; psychiatric symptoms (e.g., psychosis and personality changes, confabulation)
**GABA_A_R**	Cell surface (extra-limbic cortices)	40 yr (2 mo–88 yr); 1:1	25% (thymoma, other)	Limbic encephalitis with encephalopathy and intractable epilepsy	Behavioral and psychiatric features, including catatonia
**GABA_B_R** [73]	Cell surface (limbic system)	Men and women; 61 yr (16–77); 1.5:1	50% (SCLC)	Limbic encephalitis with intractable seizures and status epilepticus, short-term memory loss, opsoclonus-myoclonus	Features of limbic encephalitis on MRI in about 45%, psychiatric symptoms (e.g., psychosis and catatonia)
**DPPX** [74]	Cell surface (limbic system)	52 yr (13–76); 2.3:1	<10% (B-cell neoplasms)	Limbic encephalitis, encephalopathy, gastrointestinal symptoms, myoclonus, tremors, hyperekplexia	Psychiatric symptoms (e.g., psychosis, depression, delirium), PERM
**Dopamine-2R** [46]	Cell surface (basal ganglia, limbic system, and substantia nigra)	(2–15)	n/k	Basal ganglia encephalitis presenting with diverse movement disorders (e.g., dystonia, chorea, tics, parkinsonian features), psychiatric symptoms (e.g., emotional lability, depression, psychosis)	Gait disturbance, sleep disorders
**mGluR5** [45]	Cell surface (olfactory bulb, cortex, and hippocampus)	29 yr (6–75); 1.5:1	6 of 11 patients (Hodgkin’s lymphoma)	Ophelia syndrome: limbic encephalitis in association with Hodgkin lymphoma	Psychiatric symptoms, encephalopathy, myoclonus
**Neurexin-3α** [49]	Synaptic (throughout brain)	44 yr (23–57); 2:4	n/k	Infectious-like prodrome, encephalopathy, seizures, orofacial dyskinesia, cognitive decline, decrease level of consciousness, central hypoventilation	Psychiatric symptoms (less severe than those associated with NMDAR encephalitis
**IgLON5** [75]	Cell surface (brainstem and thalamus)	n/k	n/k	Non-REM and REM sleep disorders, brainstem dysfunction (e.g., bulbar symptoms, oculomotor abnormalities), gait impairment, tauopathy-associated cognitive dysfunction	Occasionally associated with severe dementia; can mimic progressive supranuclear palsy
**DNER (Tr)** [38]	Intracellular cytoplasmic (cerebellum)	n/k	>90% (Hodgkin disease)	Gait instability	Cerebellar ataxia
**P/Q type VGCC** [35]	Synaptic (cerebellum)	n/k	>90% (SCLC)	Paraneoplastic cerebellar degeneration, gait instability	Cerebellar ataxia
**mGluR1**	Cell surface (cerebellum)	n/k	A few cases (Hodgkin disease)	Gait instability	Cerebellar ataxia
**GlyR** [42]	Cell surface (brainstem and spinal cord)	49 yr (1–75); 5:1 adult	<5% (thymoma, lung, Hodgkin)	PERM, stiff-person syndrome, muscle rigidity, spasms, oculomotor disturbance, bulbar symptoms, gait impairment	Pyramidal signs, cerebellar ataxia, autonomic disturbance, excessive startle
**Amphiphysin** [39]	Intracellular synaptic (brain and spinal cord)	Exclusively Female; 60	>90% (breast cancer, SCLC)	Stiff person syndrome, confusion, memory loss	Encephalomyelitis
**Hu (ANNA-1)** [76]	Intracellular Nuclear (cerebellum and dorsal root ganglia)	63; 3:1	70 % associated with cancer	Limbic encephalitis or encephalomyelitis, painful sensory neuropathy in about 50%, cerebellar degeneration	Brainstem encephalitis (dysphagia, dysarthria, central hypoventilation)
**Yo (PCA-1)** [77]	Intracellular cytoplasmic (cerebellum)	60; 1:3	Brest and gynecological tumors	Cerebellar degeneration-associated ataxia	Vertigo, slurred speech, nystagmus, diplopia and oscillopsia
**CV2/CRMP5** [78]	Intracellular cytoplasmic (cerebellum, dorsal root ganglia, limbic system)	62; M > F	70% SCLC, 6% thymoma	Limbic encephalitis, cerebellar ataxia (26%), sensory neuropathy (47%), subacute dementia (25%)	Chorea (11%), optic neuropathy (7%)
**Ta/Ma2** [79]	Intracellular Nuclear (limbic system)	36; 4:1	Germ cell tumors (Testicular tumor)	Limbic encephalitis (64%), short-term memory impairment, REM sleep disorder	Cerebellar and brainstem dysfunction, psychiatric symptoms
**Recoverin** [80]	Intracellular cytoplasmic (retina)	65; F > M	Lung, Breast, Melanoma	Retinopathy with progressive visual loss	
**SOX-1** [81]	Synaptic (neuromuscular junction)	63; M > F	40% (SCLC)	Lambert Eaton Myasthenic syndrome	Neuropathy, paraneoplastic cerebellar degeneration
**GAD** [82,83]	Intracellular synaptic (brain and spinal cord)	41; F > M	Usually unrelated to tumor	Stiff person syndrome, limbic encephalitis	Cerebellar ataxia, intractable seizure
**GFAP** [50,84]	Astrocytes	Over age 40; slight F > M predominance	34% (ovarian teratoma among common tumors)	Meningoencephalitis, encephalitis. encephalomyelitis, encephalopathy with short-term memory loss, movement disorders	At times associated with psychiatric symptoms
**Ri (ANNA-2)** [34]	Intracellular Nuclear (cerebellum and dorsal root ganglia)	Over age 50; predominantly in F > M	SCLS, breast cancer	Encephalomyelitis, Cerebellar degeneration, stridor, laryngospasm, jaw dystonia, opsoclonus myoclonus	Sensory neuropathy, vertigo, muscle weakness

AMPA = α-amino-3-hydroxy-5-methyl-4-isoxazolepropionic acid. ANNA-1 = antineuronal nuclear antibody-type 1. CASPR2 = contactin associated protein 2. CV2/CRMP5 = CV2/Collapsin response mediator protein 5. DNER = Delta/Notch-like epidermal growth factor-related receptor. DPPX = dipeptidyl-peptidase-like protein-6. GABA = γ-aminobutyric acid. GAD = glutamic acid decarboxylase. GlyR = glycine receptor. GFAP= glial fibrillary acidic protein. IgLON = immunoglobulin G superfamily containing LSAMP, OBCAM, and Neurotrimin. LGI1= leucine-rich glioma inactivated 1. mGluR = metabotropic glutamate receptor. NMDAR = N-methyl-D-aspartate receptor. PCA-1 = Purkinje cell cytoplasmic antibody-type 1. PERM = progressive encephalomyelitis with rigidity and myoclonus. REM= rapid eye movement. SCLC = small cell lung cancer. VGCC = voltage-gated calcium channel. n/k = not known.

**Table 2:** Red flags of possible autoimmune encephalitis in individuals with unexplained neuropsychiatric disorders.

1	New-onset acute psychiatric episodes (acute mania, first episode psychosis, catatonia), particularly in those exhibiting significant adverse response (e.g., neuroleptic malignant syndrome) or resistance to antipsychotics
2	Rapidly progressive short-term memory and cognitive decline
3	Unexplained new-onset intractable epilepsy or status epilepticus, particular seizure types such as faciobrachial dystonic seizures
4	New-onset movement disorders affecting any part of the body (e.g., dyskinesias and dystonia), presentation of unclear etiology, particularly at a young age
5	Clinically significant autonomic instability
6	Deterioration, relapse, or emergence of new neurological and/or neuropsychiatric symptoms following confirmed or presumed viral illness, despite adequate treatment
7	Infectious-like prodromal illness
8	Strong personal or family history of autoimmune disorders
9	Recent diagnosis of neoplasm
10	Extreme delta brush on EEG, unexplained CSF inflammatory changes with or without oligoclonal bands (OCBs), or limbic structural abnormalities on coronal T2- or FLAIR-weighted MR imaging after excluding infectious etiologies

**Table 3:** Diagnostic criteria for possible autoimmune encephalitis.

Diagnosis can be made when all three of the following criteria have been met:
1. Subacute onset (rapid progression of less than 3 months) of working memory deficits (short-term memory loss), altered mental status, or psychiatric symptoms
2. At least one of the following: Seizures not explained by a previously known seizure disorder CSF pleocytosis (white blood cell count of more than five cells per mm3) MRI features suggestive of encephalitis New focal CNS findings
3. Reasonable exclusion of alternative causes

## Data Availability

Not applicable.
